# A 29-Year-Old Man With Type 2 Hermansky–Pudlak Syndrome and Wolff–Parkinson–White Syndrome: The Hypothesis of a Potential Link Between These Two Conditions

**DOI:** 10.1155/carm/5525411

**Published:** 2025-04-22

**Authors:** Riccardo Alcidi, Tommaso Campanella, Rosa Curcio, Lorenzo Chiatti, Alessio Arrivi, Lucia Ferranti, Giovanni Carreras, Mauro Barabani, Giacomo Pucci

**Affiliations:** ^1^Unit of Internal and Translational Medicine, Santa Maria Terni Hospital, Terni, Italy; ^2^Unit of Emergency Medicine, Santa Maria Terni Hospital, Terni, Italy; ^3^Unit of Internal and Vascular Medicine, Santa Maria Terni Hospital, Terni, Italy; ^4^Unit of Internal Medicine, Santa Margherita Della Fratta Hospital, Cortona, Italy; ^5^Unit of Cardiology, Santa Maria Terni Hospital, Terni, Italy; ^6^Unit of Internal Medicine, Degli Infermi Hospital, Narni, Italy; ^7^Department of Medicine and Surgery, University of Perugia, Perugia, Italy

## Abstract

A 29-year-old Moroccan with oculocutaneous albinism presented with a history of exertional dyspnea, recurrent epistaxis, and bacterial infections, raising suspicion of Hermansky–Pudlak syndrome (HPS). Further evaluation revealed neutropenia, impaired platelet function, pulmonary fibrosis, and mild pulmonary hypertension. An ECG identified ventricular pre-excitation with a postero-septal right accessory pathway, consistent with Wolff–Parkinson–White (WPW) syndrome. Genetic testing confirmed a homozygous mutation in the *AP3B1* gene and a diagnosis of Type 2 HPS (HPS-2) was made. HPS-2 is an extremely rare disorder, and to our knowledge, the co-occurrence of WPW syndrome has not been previously reported in literature. We propose a potential causal link between these two conditions, as mutations in the *AP3B1* gene—which encodes the beta subunit of the adapter protein 3 trafficking complex—result in mistrafficking of transmembrane proteins from the endosomal and trans-Golgi network to lysosomes and endosome–lysosome-related organelles. Specifically, the dysfunction of a transmembrane protein, namely the lysosome-associated membrane protein 2 (LAMP-2), has been implicated in the development of cardiac accessory pathways, as seen in Danon disease. We hypothesize that individuals with HPS-2 may have a genetic predisposition to WPW syndrome, and this hypothesis should be investigated in further studies.

## 1. Introduction

The Hermansky–Pudlak syndrome (HPS) is a rare autosomal recessive genetic disorder, first identified in 1959 in adults with hypopigmentation and severe haemorrhagic diathesis [1]. This disorder is characterized by interference with the function of the intracellular protein trafficking of the biogenesis of lysosome-related organelles complexes (BLOCs). The prevalence of HPS is estimated to be between 1 and 9 per 1,000,000 [2] with a significant higher prevalence (1:800) in the northwestern region of Puerto Rico, which accounts for approximately 50% of all global cases [3]. HPS is subtyped into 10 groups based on clinical features, affected genes and proteins [4]. Although there is considerable overlap between the clinical manifestations of HPS subtypes, there are important specific characteristics. For instance, Type 2 HPS (HPS-2) is characterized by neutropenia, recurrent pulmonary infections and early onset interstitial lung disease [5]. The severity of organ involvement largely determines the prognosis of HPS patients. Individuals who develop restrictive lung disease often face a life expectancy of about 10 years. However, the clinical course and survival probabilities for each subtype remain poorly described [6].

Cardiac abnormalities are uncommon in patients with HPS and, to date, no cases of Wolff–Parkinson–White (WPW) syndrome have been described in subjects with HPS. Since WPW syndrome can also arise from genetic factors that predisposes to arrythmias [7], we hypothesized a potential molecular link between these two conditions based on our observation of a WPW pattern in a patient with HPS-2.

## 2. Case Presentation

A 29-year-old Moroccan man presented to the Emergency Department for a chief complaint of acute lumbar pain radiating to the groin. On physical inspection, the patient was noted to have oculocutaneous albinism. His medical history included chronic subtle exertional dyspnea and dry cough, both of which had worsened in recent months. He also reported recurrent epistaxis, hemorrhoidal bleeding, and a tendency to bruise easily after mild injuries. The patient had also a history of spontaneous right pneumothorax and recurrent episodes of epididymitis. He denied any use of illicit drugs, tobacco, or alcohol and had no personal or family history of syncope or sudden death.

**Figure 1:**
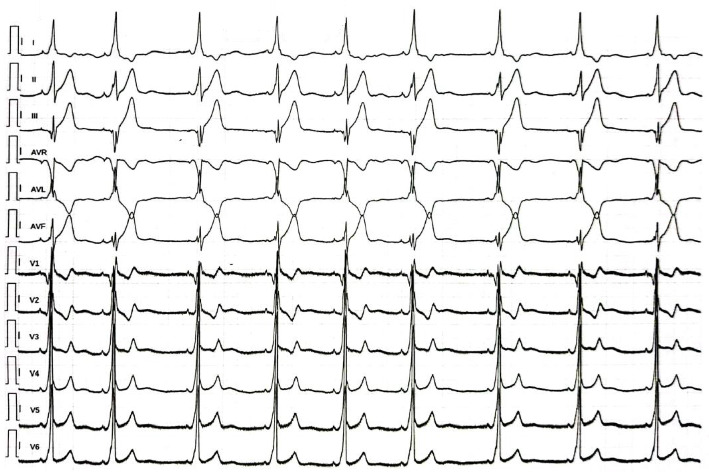
Twelve-lead ECG showing ventricular pre-excitation in postero-septal right position.

Laboratory tests showed mild leukopenia with an inversion of the leukocyte formula, characterized by neutropenia and lymphocytosis. Platelet and red blood cells count were within normal ranges. Coagulation tests were unremarkable, while platelet function test showed impaired response to agonist for platelet secretion such as collagen and thromboxane A2. An ECG revealed ventricular pre-excitation with a postero-septal right accessory pathway (Figure 1). Transthoracic 2D-echocardiography showed an increased trans-tricuspid gradient and a mildly dilated right atrium, consistent with mild pulmonary hypertension. A high-resolution chest computed tomography scan revealed signs of pulmonary fibrosis with an apical-basal gradient typical of usual interstitial pneumonia (UIP). An eye examination detected hyperopia, astigmatism, and low-grade amblyopia.

**Figure 2:**
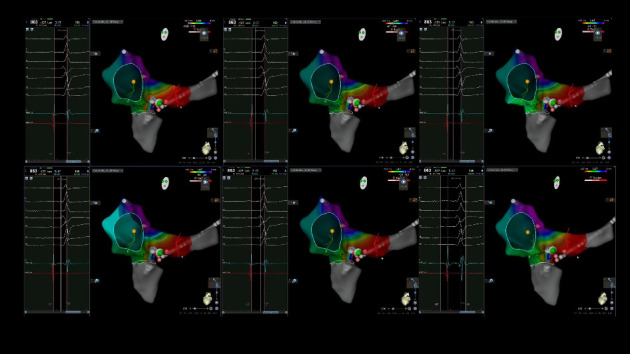
3D mapping using the carto 3 system showing ablation catheter in coronary sinus ostium and subsequent successful radiofrequency ablation.

During a lumbar spine magnetic resonance imaging (MRI), the patient experienced palpitations followed by a sudden syncopal episode, which resolved spontaneously within minutes. An electrophysiological study was subsequently performed, revealing a posterior septal right accessory pathway, consistent with WPW syndrome. The patient underwent successful transcatheter radiofrequency ablation (Figure 2). Unfortunately, he was lost to follow-up after discharge from the hospital.

Given the presence of characteristic features such as oculocutaneous albinism, pulmonary fibrosis, neutropenia with recurrent bacterial infections (such as epididymitis), and haemorrhagic diathesis, a clinical suspicion of HPS was raised. The latter has been confirmed by genetic testing, which identified a c.2757delA (p.Ile919MetfsTer8) mutation in the AP3B1 gene in homozygosity, corroborating the diagnosis of HPS-2. To the best of our knowledge, this is the first reported case of HPS-2 syndrome associated with WPW. Based on the literature, we hypothesize a potential causal link between these two conditions.

## 3. Discussion

Unlike the more common Type 1, HPS-2 is extremely rare [8]. HPS-2 is characterized by severe static, G-CSF responsive, congenital neutropenia, and interstitial lung disease. Globally, there are approximately 1200 documented cases of HPS [6], with around 35 individuals with HPS-2 reported in literature [9].

HPS-2 is caused by mutations that inactivate the *AP3B1* gene, which encodes the beta subunit of the adapter protein 3 (AP3) trafficking complex. AP3 is a ubiquitously expressed heterotetrameric complex, responsible for sorting transmembrane proteins from the endosomal and trans-Golgi network to lysosomes and endosome–lysosome-related organelles, such as melanosomes, platelet-dense bodies, antigen-processing compartments, and azurophil lytic granules [10]. Examples of transmembrane proteins include CD63, lysosome-associated membrane protein 1 (LAMP-1), lysosome-associated membrane protein 2 (LAMP-2), tyrosinase, CD1b, and neutrophil elastase. The mistrafficking of these proteins explains the associated signs and symptoms of HPS-2. For instance, the mistrafficking of neutrophil elastase is linked to neutropenia, as neutrophil elastase is crucial for the differentiation of myeloid progenitors into mature neutrophils [11]. Similarly, the mistrafficking of platelets dense bodies, which store ADP, ATP, serotonin, calcium, and phosphate, impairs their release, thereby hindering platelet aggregation [12].

WPW syndrome, with a prevalence of approximately 1 in 2500 individuals in the age range 25–30 years [13], is a cardiac syndrome that has been rarely linked to genetic defects. However, several genes have been proposed as potential candidates for inherited forms of WPW syndrome, often in conjunction with other manifestations of cardiomyopathy [7]. Some proposed potential mechanisms for the proarrhythmogenic phenotype involve thinning of the atrioventricular border (annulus fibrosus) due to excessive glycogen storage. Whether WPW develops as a secondary process in systemic diseases with pulmonary hypertension, or if it acts as a contributing factor, this remains uncertain. This hypothesis has been first proposed in a case report of a man with systemic sclerosis sine scleroderma, interstitial lung disease, and WPW syndrome [14]. However, this remains the only documented case to date. This area remains a subject of research and is still under investigation.

Interestingly, a WPW pattern was found at the ECG of a cohort of 41 patients with Danon disease, a rare X-linked myopathy caused by a defect in LAMP-2. A loss-of-function mutation in LAMP-2 gene disrupts autophagy by impairing the fusion between vacuoles and lysosomes, ultimately leading to vacuolar myopathy [15]. There is a growing hypothesis that LAMP-2 dysfunction may affect the development of the annulus fibrosus, thus contributing to the development of anterograde or retrograde (or both) accessory pathways. This hypothesis is supported by electrophysiological studies (EPSs) in patients with Danon disease [16] although it requires further confirmation.

Based on our observation in a patient with HPS-2 syndrome, we hypothesize that the genetic deficiency of the beta subunit of the AP3 trafficking complex, and the resulting disruption of the transmembrane proteins sorting from the endosomal and trans-Golgi network to the lysosomes, may lead to LAMP-2 dysfunction-mediated defective autophagy. This could represent the molecular basis for the development of the WPW syndrome.

Other potential cellular mechanisms involved in familial WPW could imply alterations in the properties of cardiac ion channels, such as the voltage-gated sodium channel, or mutations in the PRKAG2 gene, which encodes the regulatory *γ*2-subunit of the AMP-activated protein kinase (AMPK) [17]. Mutations in PRKAG2 have been linked to ultrastructural changes associated with AP3, potentially leading to mitochondrial dysfunction. This dysfunction has been associated with increased AMPK phosporylation, as it was found in HPS-2 cells [18]. This heightened AMPK activity may contribute to glycogen accumulation in anatomical structures regulating the atrioventricular conduction [19].

Given the rarity of both conditions, confirming or refusing such hypothesis through an epidemiological approach may be challenging and, at present, these scenarios remain speculative. Therefore, we suggest that individuals diagnosed with HPS-2 should be screened for accessory pathways, potentially through EPS, to identify any underlying cardiac conduction abnormalities.

## 4. Disclosures

The patient gave the informed consent to undergo diagnostic and therapeutic exams in accordance with the institution's procedures. A written informed consent was obtained before each invasive procedure. The patient gave witnessed informed consent for the publication of the case report, provided that all details that would enable any reader to identify the person are omitted.

## Data Availability

No datasets were generated or analyzed during the current study.

## References

[1] Hermansky F., Pudlak P. (1959). Albinism Associated with Hemorrhagic Diathesis and Unusual Pigmented Reticular Cells in the Bone Marrow: Report of Two Cases With Histochemical Studies. *Blood*.

[2] Introne W. J., Huizing M., Malicdan M. C. V., Adam M. P., Feldman J., Mirzaa G. M. (2000). Hermansky-Pudlak Syndrome. *GeneReviews®*.

[3] Vicary G. W., Vergne Y., Santiago-Cornier A., Young L. R., Roman J. (2016). Pulmonary Fibrosis in Hermansky-Pudlak Syndrome. *Annals of the American Thoracic Society*.

[4] De Jesus Rojas W., Young L. R. (2020). Hermansky-Pudlak Syndrome. *Seminars in Respiratory and Critical Care Medicine*.

[5] Hengst M., Naehrlich L., Mahavadi P. (2018). Hermansky-Pudlak Syndrome Type 2 Manifests With Fibrosing Lung Disease Early in Childhood. *Orphanet Journal of Rare Diseases*.

[6] El-Chemaly S., Young L. R. (2016). Hermansky-Pudlak Syndrome. *Clinics in Chest Medicine*.

[7] Vătășescu R. G., Paja C. S., Şuș I., Cainap S., Moisa Ş M., Cinteză E. E. (2024). Wolf-Parkinson-White Syndrome: Diagnosis, Risk Assessment, and Therapy-An Update. *Diagnostics*.

[8] Huizing M., Scher C. D., Strovel E. (2002). Nonsense Mutations in ADTB3A Cause Complete Deficiency of the β3A Subunit of Adaptor Complex-3 and Severe Hermansky-Pudlak Syndrome Type 2. *Pediatric Research*.

[9] Yokoyama T., Gochuico B. R. (2021). Hermansky-Pudlak Syndrome Pulmonary Fibrosis: A Rare Inherited Interstitial Lung Disease. *European Respiratory Review*.

[10] Fontana S., Parolini S., Vermi W. (2006). Innate Immunity Defects in Hermansky-Pudlak Type 2 Syndrome. *Blood*.

[11] Berliner N., Horwitz M., Loughran T. P. (2004). Congenital and Acquired Neutropenia. *Hematology*.

[12] Dell’Angelica E. C., Shotelersuk V., Aguilar R. C., Gahl W. A., Bonifacino J. S. (1999). Altered Trafficking of Lysosomal Proteins in Hermansky-Pudlak Syndrome Due to Mutations in the Beta 3A Subunit of the AP-3 Adaptor. *Molecular Cell*.

[13] Lu C. W., Wu M. H., Chen H. C., Kao F. Y., Huang S. K. (2014). Epidemiological Profile of Wolff-Parkinson-White Syndrome in a General Population Younger Than 50 Years of Age in an Era of Radiofrequency Catheter Ablation. *International Journal of Cardiology*.

[14] Park Y., Woo H., Yoon H. (2007). Systemic Sclerosis Sine Scleroderma Associated With Wolff-Parkinson-White Syndrome. *Scandinavian Journal of Rheumatology*.

[15] Cenacchi G., Papa V., Pegoraro V., Marozzo R., Fanin M., Angelini C. (2020). Review: Danon Disease: Review of Natural History and Recent Advances. *Neuropathology and Applied Neurobiology*.

[16] Chen X., Fu L., He J. (2022). A Frequent Observation of Wolff-Parkinson-White Syndrome and Fasciculoventricular Pathways in Patients With Danon Disease. *Circulation Journal*.

[17] Light P. E. (2006). Familial Wolff-Parkinson-White Syndrome: A Disease of Glycogen Storage or Ion Channel Dysfunction?. *Journal of Cardiovascular Electrophysiology*.

[18] Cuevas-Mora K., Roque W., Shaghaghi H. (2021). Hermansky-Pudlak Syndrome-2 Alters Mitochondrial Homeostasis in the Alveolar Epithelium of the Lung. *Respiratory Research*.

[19] Porto A. G., Brun F., Severini G. M. (2016). Clinical Spectrum of PRKAG2 Syndrome. *Circulation: Arrhythmia and Electrophysiology*.

